# Relationship between comb development, immune regulation, growth hormone, testosterone, and growth traits in Tianfu broilers

**DOI:** 10.1016/j.psj.2025.105367

**Published:** 2025-05-28

**Authors:** Kunlong Qi, Felix Kwame Amevor, Zheliang Liu, Juan He, Dan Xu, Chencan Zhai, Yingjie Wang, Liuting Wu, Yan Wang, Gang Shu, Xiaoling Zhao

**Affiliations:** a State Key Laboratory of Swine and Poultry Breeding Industry, College of Animal Science and Technology, Sichuan Agricultural University, Chengdu, Sichuan, PR China; b Farm Animal Genetic Resources Exploration and Innovation Key Laboratory of Sichuan Province, College of Animal Science and Technology, Sichuan Agricultural University, Chengdu, Sichuan, PR China; c Key Laboratory of Livestock and Poultry Multi-omics, Ministry of Agriculture and Rural Affairs, Sichuan Agricultural University, Chengdu, Sichuan, PR China; d Department of Basic Veterinary Medicine, Sichuan Agricultural University, Chengdu, Sichuan, PR China

**Keywords:** Tianfu broiler, Comb size, Growth hormone, Testosterone, Correlation analysis

## Abstract

The comb, a secondary sexual characteristic in chickens, plays a crucial role in sexual selection, physiological regulation, and growth performance. This study explores the relationship between comb development, circulating hormone levels, and growth traits in Tianfu broilers. Weekly measurements of comb size and body weight from hatching to market age (day 70) revealed significant individual variations in comb traits. Based on comb size, chickens were categorized into large comb and small comb groups at market age. Histological analysis revealed that chickens with large combs exhibited thicker dermal, but the central layer exhibits the opposite, with sex-specific differences in collagen fiber content and epithelium thickness. Additionally, while only a few lymphocytes were observed in the combs of the large-comb group, focal lymphocyte aggregation was evident in the small-comb group. Circulating growth hormone (**GH**) and testosterone levels were significantly higher in chickens with large combs, particularly in roosters, where testosterone levels showed a significant correlation with testicular weight. It was observed that the GH levels were significantly correlated with comb development, independent of sex. Correlation analysis indicated a trade-off between comb size and carcass yield, suggesting that resource allocation favors ornamentation and reproductive system over meat production. These findings provide insights into the biological significance of comb development, emphasizing its potential as a marker for reproductive fitness and immunomodulatory functions in poultry breeding.

## Introduction

Secondary sexual characteristics in animals, such as plumage coloration and comb size in birds, serve as indicators of reproductive fitness and also reflect the underlying physiological processes that influence growth and immune function (Cornwallis et al., 2014; Łukaszewicz, et al., 2018). In poultry, particularly chickens, the comb is a conspicuous secondary sexual trait that has received increasing attention due to its multifaceted biological roles, encompassing sexual selection, thermoregulation, and endocrine signaling (Johnsson, et al., 2014; Nelson, et al., 1995; Younis, et al., 2023). While early studies primarily emphasized the comb’s role in mate choice and evolutionary advantage (Cornwallis, et al., 2014; Imsland, et al., 2012), there is now growing recognition of its potential as a biomarker for production performance and systemic health.

Comb development is modulated by a complex interplay of genetic, hormonal, and environmental factors (Johnsson, et al., 2014; Younis, et al., 2023). Recent studies have shown that the growth of secondary sexual traits, including the comb, is significantly influenced by circulating levels of sex steroids and growth-related hormones such as testosterone and growth hormone (**GH**) (Lengyel, et al., 2024). Testosterone, in particular, has been implicated in promoting comb growth while concurrently exerting immunosuppressive effects, underscoring a trade-off between reproductive investment and immune competence (Folstad and Karter, 1992; Lengyel, et al., 2024; Verhulst, et al., 1999). This phenomenon, termed the immunocompetence handicap hypothesis, suggests that ornamentation in males is costly and can only be maintained by individuals in superior physiological condition. Moreover, comb size has been positively correlated with slaughter weight, and reproductive system, making it a potentially valuable phenotypic predictor for growth selection in broilers (Cornwallis, et al., 2014; Imsland, et al., 2012). In roosters, large combs have been associated with reduced circulating lymphocytes (Zuk, et al., 1995). However, despite these associations, the mechanistic pathways linking comb development with immune modulation, hormonal regulation, and economically important traits such as growth rate and carcass quality remain inadequately understood.

Tianfu broilers, a native Chinese breed renowned for their rapid growth and robust phenotype, offer a unique model for studying the co-regulation of ornamental and production traits. Their pronounced variation in comb morphology provides an excellent opportunity to investigate how comb size correlates with circulating testosterone and GH levels, immune parameters, skeletal growth, reproductive system, and slaughter traits. Hence, addressing these associations can provide critical insights into the physiological costs and benefits of ornamental trait expression in modern poultry production systems. Therefore, the present study aims to elucidate the biological interconnections among comb development, hormonal regulation, immune function, and growth traits in Tianfu broilers. By integrating morphometric, endocrinological, and phenotypic data, this study seeks to fill key gaps in current knowledge and advance our understanding of how secondary sexual traits reflect and potentially predict performance outcomes in poultry breeding programs.

## Materials and methods

### Animals and tissues

The Tianfu broilers were provided by Sichuan Banghe Agricultural Technology Co., Ltd. From hatching (day 0) to market age (day 70), the chicken’s comb size was measured weekly. At market age, the chickens were euthanized for sample collection. The animal experiment was approved by the Institutional Animal Care and Use Committee of Sichuan Agricultural University (SYXK2019-187). All experiments were conducted in accordance with the guidelines provided by the Animal Welfare and Ethics Committee of Sichuan Agricultural University, China.

### Measurement of comb and body weight development

From hatching to market age, the comb size and body weight of Tianfu broilers were recorded weekly. To evaluate growth performance based on comb size, comb length and comb height were measured at market age for all chickens. The comb area was calculated as the product of comb length and comb height (Grethen, et al., 2024; Mukhtar and Khan, 2012; Qi, et al., 2025). Thresholds values for categorizing chickens into the large and small comb groups were determined based on the maximum, minimum, and median comb areas within the population (Table 1). Ten chickens were randomly selected from each group within the specified threshold range for further measurement.

**Table 1:** Selection of threshold values for grouping large and small combs (mm^2^).

**Term**	**Rooster**	**Hen**
max	3320.76	1717.09
median	2122.21	711.34
min	779.34	228.33
threshold for large comb group	2721.48	1214.21
threshold for small comb group	1450.77	469.83

At 70 days of age, comb length and height were measured for the entire flock, and comb area was calculated accordingly (*n* = 50). The comb area was then used to group the Chickens were then group based comb area extremes. For the large comb group: individuals with comb areas between (maximum − median)/2+ median and the maximum value were selected. For the small comb group: individuals with comb areas between the minimum and (minimum + median)/2 were selected. From the population within these threshold ranges, 10 individuals were randomly selected from each of the large and small comb groups to measure growth performance.

### Chicken circulating GH and testosterone ELISA assay

Circulating levels of GH and testosterone were analyzed using enzyme-linked immunosorbent assay (**ELISA**). Circulating levels of GH (MM-1609O1) and testosterone (MM-0786O1) were quantified using ELISA kits, following the manufacturer’s instructions (Jiangsu Meimian Industrial Co., Ltd, China). Thereafter, the absorbance was measured at 450 nm using the microplate spectrophotometer.

### Histological observation of the comb tissue using H&E staining

Chicken comb tissue was fixed in 4 % paraformaldehyde and embedded in paraffin for histological analysis. The tissues were sectioned at 5 μm thickness and mounted on glass slides. Following deparaffinization and rehydration, the sections were stained with hematoxylin for 30 minutes, followed by counterstaining with eosin for 5 minutes. The sections were then examined under an optical microscope (DM1000, Leica, Germany).

### Masson’s trichrome staining of comb tissue

For collagen staining, sections were prepared similarly to H&E staining. After deparaffinization, the sections were stained with hematoxylin for 2-5 minutes, rinsed, differentiated with hydrochloric acid alcohol, and blued with ammonia water. Next, the sections were stained with ponceau acid fuchsin for 5-10 minutes and washed with distilled water. The sections were then treated with 1 % phosphomolybdic acid for 1-3 minutes before staining with aniline blue solution for 5 minutes. After a final wash, the sections were dried at 60°C, cleared with xylene, and mounted with neutral resin. Thereafter, the sections were examined using an optical microscopy (DM1000, Leica, Germany).

### Statistical analysis

The data were expressed as mean ± standard error of the mean (**SEM**). Statistical analysis was performed using IBM SPSS (version 27) software, tissue section measurements and statistics were conducted using ImageJ software, and visualization was carried out using ggplot2 package (Ginestet, 2011), and the Mantel test was performed using the linkET package of R (version 4.4.1).

## Results

### Growth patterns and characteristics of body weight and comb development in Tianfu broilers

**Fig. 1:**
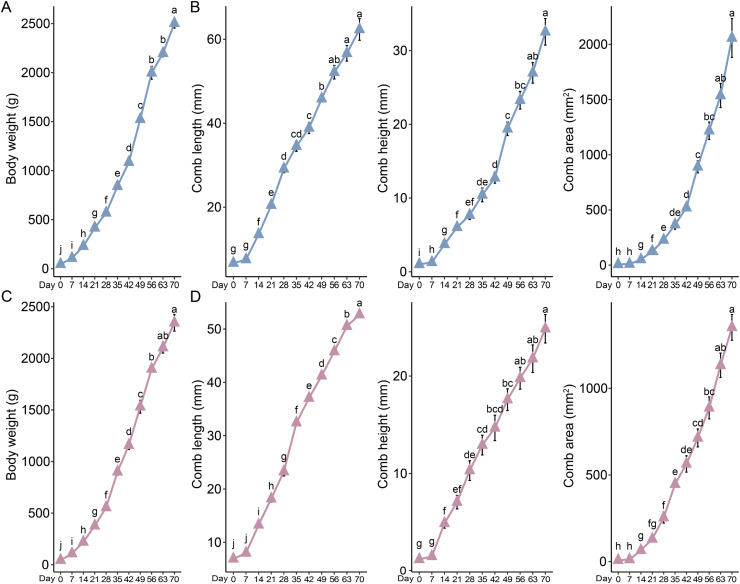
Changes in body weight and comb traits of Tianfu broilers from hatching (day 0) to market age (day 70). (A) Changes in body weight of roosters, (B) Changes in comb length, comb height, and comb area with age. Statistical analysis was performed using one-way ANOVA, followed by Tukey’s or Dunnett’s multiple comparison test (*n* = 10). Different letters indicate statistically significant differences (*P* < 0.05).

By the time they reach market age, both roosters and hens exhibit similar growth and development patterns in body weight and comb size, characterized by slow initial growth followed by a gradual acceleration. Specifically, the body weight of roosters increases rapidly from hatch to 70 days of age (Fig. 1A), while hens experience a slowdown in weight gain starting at 56 days of age (Fig. 1C). Regardless of sex, the development of comb length, height, and area progresses slowly during the first 7 days, then accelerates. However, in roosters, this development slows down again starting from 49 days of age (Fig. 1B). For hens, the comb length grows relatively quickly, while the comb height shows a slower increase starting from 42 days (Fig. 1D).

To explore the differences in comb development between roosters and hens, we analyzed the impact of sex. First, we found that for body weight, there was a difference only at 21 days of age during the 0-70 days period (Fig. S1A). Starting from 21 days of age (except for 35 and 42 days), the comb height of roosters became significantly greater than that of hens. Our findings also revealed that before 42 days of age, hens exhibited greater comb height than roosters. However, after this point, roosters’ comb height surpassed that of hens. Likewise, the comb area in roosters was not significantly larger than that of hens until 49 days of age (Fig. S1B).

### Differences in skeletal development and slaughter traits in Tianfu broilers with different comb sizes

Interestingly, we observed considerable variation in comb size among both roosters and hens during this process. To assess the influence of comb size on chicken development, we classified the chickens into two groups based on comb size. The results indicated that chickens with large combs had significantly greater testicular or ovary weight and testicular or ovary index than those with small combs. Surprisingly, the eviscerated weight ratio was significantly lower in roosters with large combs compared to small combs, whereas no such difference was observed in the hens (Table 2). On the other hand, no differences in skeletal development were observed between the large comb and small comb groups, regardless of whether they were roosters or hens (Table S1).

**Table 2:** Analysis of comb, slaughter traits of Tianfu broilers with different comb sizes.

		**Rooster**			**Hen**	
**Group**	**Large**	**Small**	***P*-value**	**Large**	**Small**	***P*-value**
Comb weight	9.19 ± 0.88	2.17 ± 0.22	***	1.17 ± 0.14	0.37 ± 0.04	***
Comb length	78.42 ± 1.10	53.51 ± 1.54	***	49.54 ± 0.50	33.12 ± 0.51	***
Comb height	37.38 ± 0.54	21.91 ± 0.81	***	25.02 ± 0.17	11.12 ± 0.42	***
Comb area	2933.71 ± 73.86	1179.52 ± 67.77	***	1229.91 ± 5.10	352.11 ± 19.39	***
Testicular/ovary weight	8.49 ± 1.34	1.19 ± 0.14	***	1.08 ± 0.06	0.85 ± 0.07	*
Slaughter ratio	87.35 ± 0.56	87.83 ± 1.12	ns	88.95 ± 0.33	88.25 ± 0.25	ns
Half-eviscerated weight ratio	81.94 ± 1.02	81.91 ± 0.96	ns	81.58 ± 0.56	81.21 ± 0.42	ns
Eviscerated weight ratio	63.95 ± 0.22	66.61 ± 0.97	*	67.81 ± 0.18	67.71 ± 0.92	ns
Breast muscle weight ratio	17.27 ± 0.91	17.41 ± 0.98	ns	18.26 ± 0.39	18.61 ± 0.48	ns
Leg muscle weight ratio	21.26 ± 0.46	20.65 ± 0.44	ns	19.94 ± 0.50	20.05 ± 0.35	ns
Testicular/ovary index	0.37 ± 0.06	0.07 ± 0.01	***	0.06 ± 0.00	0.04 ± 0.00	*

Comparisons between different comb sizes were performed using independent sample t-tests, *n* = 10. * indicates *P* < 0.05, ** or *** indicates *P* < 0.01, and ns indicates no significance.

### Structural characteristics and circulating GH and testosterone profiles of Tianfu broilers with different comb sizes

**Fig. 2:**
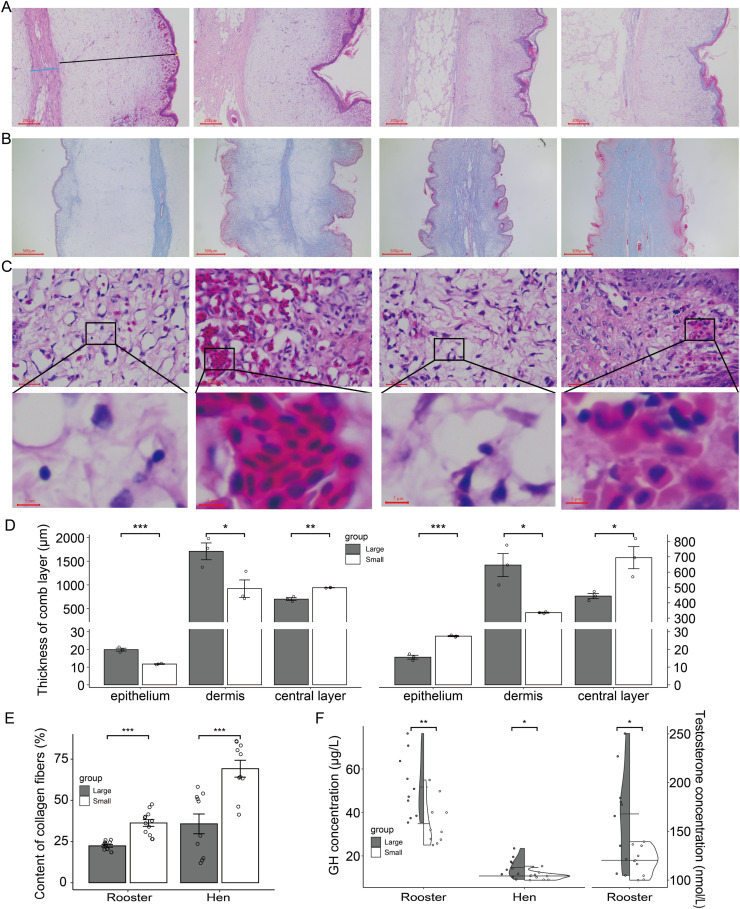
Structural characteristics of comb and circulating hormone levels in Tianfu broilers with different comb sizes at 70 days of age. (A) Representative images of comb H&E staining (*n* = 3). Each pair of images forms a group: the first group represents roosters, and the second represents hens. In each group, the first image shows large comb, and the second shows small comb. The yellow, black, and blue line areas represent the epithelium, dermis, and central layer, respectively. Scale bar = 200 μm. (B) Representative images of comb Masson staining (*n* = 3). Each pair of images forms a group: the first group represents roosters, and the second group represents hens. In each group, the first image shows large combs, and the second shows small combs. Scale bar = 500 μm. (C) Representative images of comb H&E staining (*n* = 3). Each pair of images forms a group: the first group represents roosters, and the second group represents hens. In each group, the first image shows large comb, and the second shows small comb. Scale bar = 20 μm (up), 5 μm (down). (D) Thickness of different comb layers, (E) collagen fiber content, and (F) Circulating GH levels in chickens with different comb sizes (left panel) and circulating testosterone levels in roosters with different comb sizes (right panel) (*n* = 10). Independent samples t-tests were used for comparisons between different comb sizes. Statistical significance was indicated as follows: * *P* < 0.05, ** *P* < 0.01, *** *P* < 0.001.

To further investigate the differences in structural characteristics, immune capacity, and hormone levels between combs of different sizes, we found significant variations in the thickness of different comb layers. Specifically, the dermis of the comb was noticeably thicker in the large comb group, while the central layer is relatively thinner, regardless of whether they were roosters or hens. For roosters with large combs, the epithelium thickness was significantly greater than that of the small comb group, whereas the opposite was true for hens (Fig. 2A and D). Collagen fibers, which provide elasticity and resilience to the comb and play a role in support and tissue repair, were significantly elevated in the small comb group (Fig. 2B and E).

Moreover, we found a small number of lymphocytes in the combs of roosters from the large comb group, while inflammatory cell aggregation was observed in the combs of the small comb group. Interestingly, a similar phenomenon was observed in hens: a small number of lymphocytes were present in the combs of the large comb group, while focal aggregation was found in the small comb group (Fig. 2C). Additionally, to determine whether circulating GH and testosterone levels differed between chickens with different comb sizes, we conducted ELISA tests. The results showed that GH levels were higher in the large comb group for both roosters and hens. As expected, testosterone levels were significantly higher in the large comb group of roosters compared to the small comb group (Fig. 2F).

### Correlation analysis of circulating GH and testosterone, comb development, skeletal growth, reproductive system, and slaughter traits

**Fig. 3:**
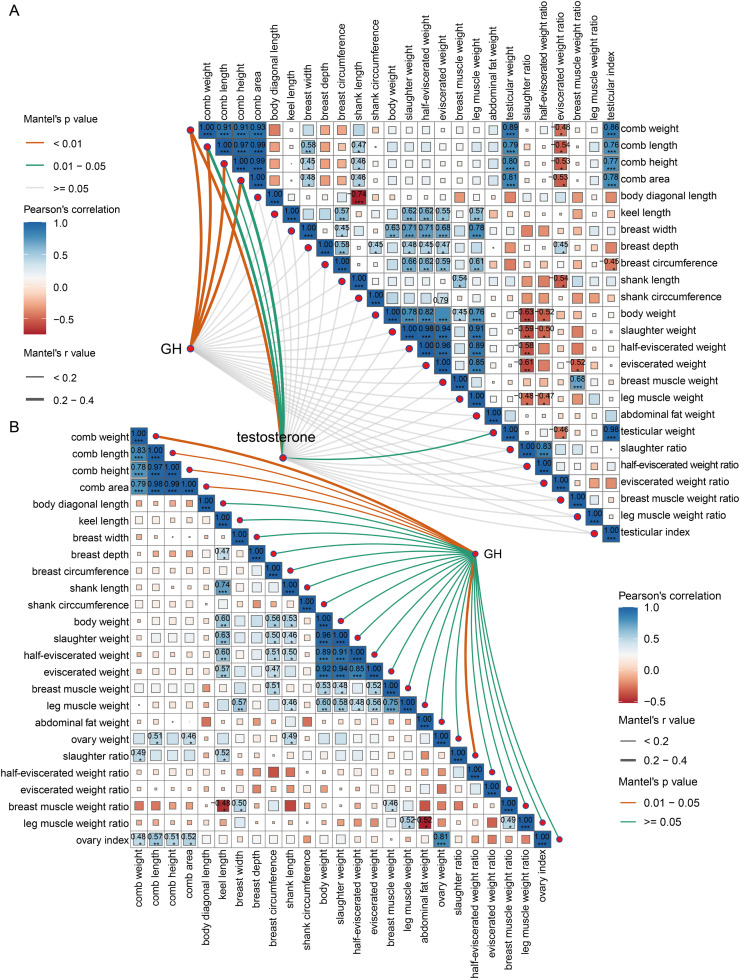
Mantel test of circulating GH and testosterone with comb traits, skeletal development, and slaughter traits in Tianfu broilers. (A) Mantel test analysis of circulating GH and testosterone with comb development, skeletal development, and slaughter traits in roosters. (B) Mantel test analysis of circulating GH with comb development, skeletal development, and slaughter traits in hens. The Mantel test analysis was performed using the linkET package in R. Statistical significance in the correlation heatmap was represented as follows: * *P* < 0.05, ** *P* < 0.01, *** *P* < 0.001.

To further explore the relationship between hormone levels and comb development, growth performance, we conducted a correlation analysis. The results showed that in roosters, both GH and testosterone were significantly correlated with comb development. Moreover, testosterone was significantly correlated with testicular weight. Furthermore, we found that comb development was significantly positively correlated with breast width, shank length, testicular weight, and testicular index, while it was significantly negatively correlated with eviscerated weight ratio (Fig. 3A). For hens, GH was also significantly correlated with comb development, as well as with slaughter ratio. We also observed a phenomenon in hens similar to that in roosters: comb development was significantly positively correlated with ovary weight and ovary index (Fig. 3B).

## Discussion

The study of comb development provides valuable insights into avian sexual differentiation, reproductive capacity, and overall health while serving as an important model for understanding gene regulation, hormonal influences, and the intricate relationship between phenotype and genetics (Cornwallis and Birkhead, 2007; Holt, et al., 2024; Ross, et al., 2020). In addition, comb size and morphology are widely recognized as indicators of reproductive performance and growth traits in chickens, contributing to improved selection accuracy and farming efficiency (Parker, 2003). In this study, we investigated the relationship between comb development, growth performance, and reproductive system in Tianfu broilers, to reveal the multifaceted impact of comb characteristics on poultry production.

Histological analysis revealed significant structural differences between combs of varying sizes, particularly in dermis and central layers thickness. Moreover, roosters with large combs exhibited a thicker epithelium, whereas hens displayed the opposite pattern, suggesting sex-specific variations in comb structure and function. These structural differences may represent adaptive strategies that balance ornamental expression with physiological or immune requirements (Vergara and Martínez-Padilla, 2012; Verhulst, et al., 1999). Comb size is also a reliable indicator of hormonal status and overall chicken health (Fölsch, et al., 1994; Mougeot, et al., 2005). Previous studies have shown that larger combs are associated with elevated circulating levels of GH and testosterone, particularly in roosters, supporting the notion that hormonal regulation plays a central role in comb development (Fennell and Scanes, 1992). Testosterone, in particular, shows a strong positive correlation with testicular weight, reinforcing its dual role in driving secondary sexual trait expression and enhancing reproductive capacity. However, this association may incur immunological costs, suggesting a potential trade-off between ornamental development and immune function (Chen, et al., 2009; Verhulst, et al., 1999). Given the preliminary evidence linking comb development to immune function, lymphocytes play a critical role in maintaining normal immune regulation (Baumann, et al., 2021; Vezzoli, et al., 2016). Our findings also revealed that immune cells in the combs of chickens in the large comb group were diffusely distributed, whereas inflammatory cell aggregation was more prevalent in the small comb group. This suggests that comb size could serve as a proxy for immune competence in poultry (Baratti, et al., 2010; Dunn, et al., 2013).

Correlation analysis further emphasized the biological significance of comb development. Interestingly, comb size was positively correlated with reproductive organ development (example testicular and ovary weight) but negatively correlated with eviscerated weight ratio, particularly in roosters (McGary, et al., 2003). This suggests a trade-off in resource allocation, where energy devoted to ornamentation and reproductive system may come at the expense of meat production efficiency (Fennell, et al., 1996; Stokkan, et al., 1986). In roosters, comb traits exhibited a positive correlation with skeletal growth (example breast width and shank length), indicating a potential link between ornamentation and overall growth performance (Dong, et al., 2019). However, the negative correlation between comb size and carcass yield highlights the need for careful consideration of these trade-offs in poultry breeding programs (Holt, et al., 2024). In hens, the correlation between GH levels and comb development, as well as slaughter ratio suggests that comb traits could serve as useful indicators of growth efficiency and reproductive potential (Bowen, et al., 1987). Further studies should investigate the genetic and environmental factors influencing comb development to better understand its evolutionary and practical relevance in poultry production.

## Conclusion

The results from this study, highlight the intricate relationship between comb development, hormonal regulation, and growth traits in Tianfu broilers. The study demonstrated that large combs were associated with enhanced reproductive organ development and higher circulating levels of GH and testosterone, particularly in roosters, reinforcing the pivotal role of these hormones in ornamentation and reproductive capacity. Furthermore, the observed trade-offs between comb size, immune function, and carcass yield emphasize the need to balance ornamental traits with production efficiency in poultry breeding programs. These findings deepen our understanding of the biological relationship between comb development and individual growth performance, offering a theoretical foundation for advancing precision breeding strategies in poultry. Future studies should investigate the genetic and molecular pathways underlying comb morphology and its association with growth and immune traits, while also evaluating how these traits interact with environmental factors and the long-term consequences of comb-based selection on production performance across different chicken breeds and rearing systems.

## Ethics approval and consent to participate

The animal experimental procedures were approved by the Institutional Animal Care and Use Committee of Sichuan Agricultural University, China (2022. 12. 06. Certification No. SYXK2019-187), and all the experiments were conducted in accordance with the guidelines provided by the Sichuan Agricultural University Laboratory Animal Welfare and Ethics.

## CRediT authorship contribution statement

K.Q, F.K.A, Z.L, and X.Z: Conceptualization, Data curation, Formal analysis, Investigation, Methodology, Software, Writing - original draft, and Writing-review & editing; C.Z and J.H: Formal analysis, Methodology, Writing - review & editing, Software; Ya.W, D.X, Y.W, L.W and G.S: Formal analysis, Writing-review & editing, Resources, Software; and Methodology; X.Z: Funding acquisition, Supervision, and Validation. All authors have read and agreed to the published version of the manuscript.

## Declaration of competing interest

The authors declare that they have no known competing financial interests or personal relationships that could have appeared to influence the work reported in this paper.

## Appendix Supplementary materials

Image, application 1
