# Supplementary material for: Relationship between comb development, immune regulation, growth hormone, testosterone, and growth traits in Tianfu broilers
Source: Poult Sci. 2025 May 28;104(8):105367. doi: 10.1016/j.psj.2025.105367 (PMC12173053; doi:10.1016/j.psj.2025.105367)
Supplement: Supplementary file 1 [file mmc1.docx]

**Running Title: Comb, Immunity, and Hormones in Broilers**

**Relationship Between Comb Development, Immune Regulation, Growth Hormone, Testosterone, and Growth Traits in Tianfu Broilers**

Kunlong Qi^†,‡,§,1^, Felix Kwame Amevor^†,‡,§,1^, Zheliang Liu^†,‡,§^, Juan He^†,‡,§^, Dan Xu^†,‡,§^, Chencan Zhai^†,‡,§^, Yingjie Wang^†,‡,§^, Liuting Wu^†,‡,§^, Yan Wang^†,‡,§^, Gang Shu^#^, Xiaoling Zhao^†,‡,§,*^

^†^State Key Laboratory of Swine and Poultry Breeding Industry, College of Animal Science and Technology, Sichuan Agricultural University, Chengdu, Sichuan, P. R. China.

^‡^Farm Animal Genetic Resources Exploration and Innovation Key Laboratory of Sichuan Province, College of Animal Science and Technology, Sichuan Agricultural University, Chengdu, Sichuan, P. R. China.

^§^Key Laboratory of Livestock and Poultry Multi-omics, Ministry of Agriculture and Rural Affairs, Sichuan Agricultural University, Chengdu, Sichuan, P. R. China.

^#^Department of Basic Veterinary Medicine, Sichuan Agricultural University, Chengdu, Sichuan, P. R. China.

^1^These authors contributed equally to this work.

***Corresponding Author:** Xiaoling Zhao, Farm Animal Genetic Resources Exploration and Innovation Key Laboratory of Sichuan Province, Sichuan Agricultural University, Chengdu, Sichuan, China. Zipcode: 611130.

E-mail: [zhaoxiaoling@sicau.edu.cn](mailto:zhaoxiaoling@sicau.edu.cn)

**Supplementary Figures and tables**


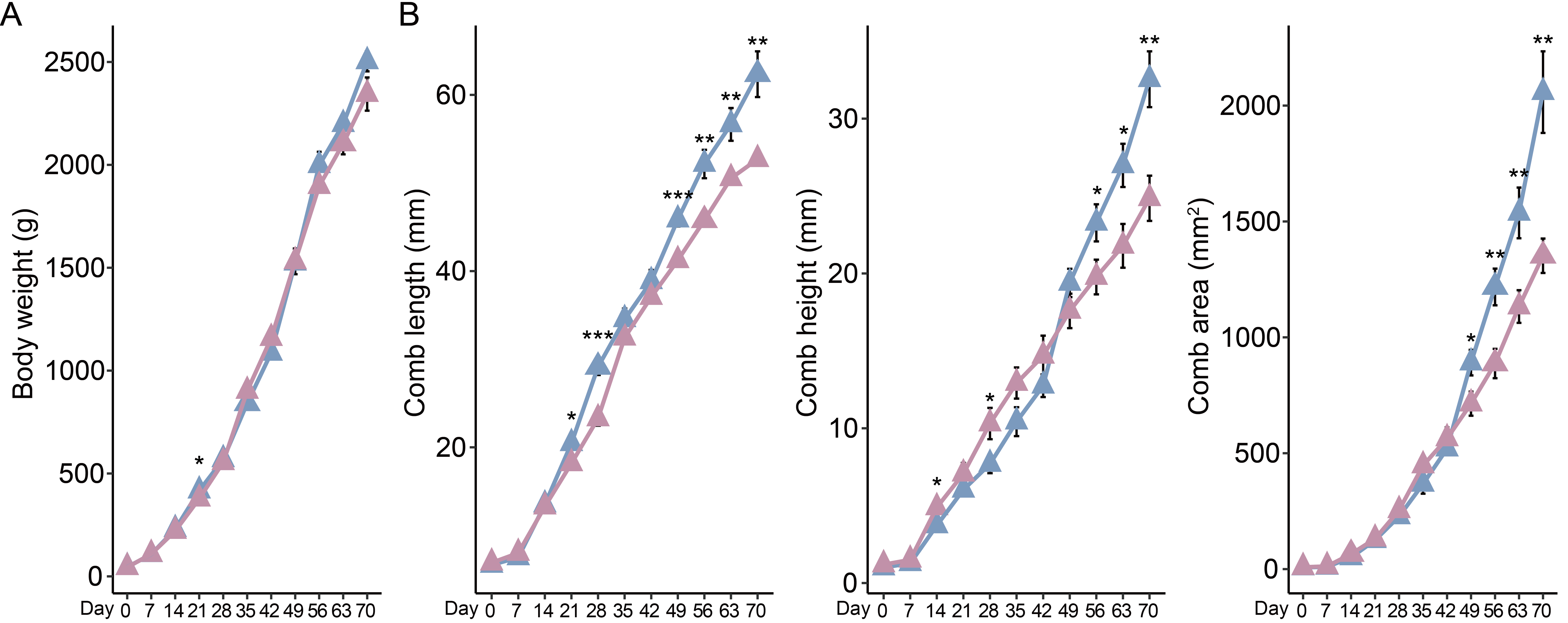


**Figure S1.** Changes in body weight and comb traits between males and females Tianfu broilers from hatching to market age. (A) Changes in body weight; (B) Changes in comb length, comb height, and comb area with age. Comparisons between different comb sizes were performed using independent sample t-tests. * indicates *P*<0.05, ** indicates *P*<0.01, *** indicates *P*<0.001, and unmarked indicates no significance, n = 10.

**Table S1** Analysis of comb, skeletal development, slaughter performance of different comb sizes of Tianfu broilers

|  |  | **Rooster** |  |  | **Hen** |  |
| --- | --- | --- | --- | --- | --- | --- |
| **Group** | **Large** | **Small** | ***P*-value** | **Large** | **Small** | ***P*-value** |
| Chest circumference | 26.83±0.35 | 27.58±0.49 | ns | 27.08±0.39 | 26.76±0.39 | ns |
| Shank length | 127.68±2.18 | 122.63±1.66 | ns | 108.76±1.20 | 107.95±2.03 | ns |
| Shank circumference | 5.11±0.12 | 5.04±0.08 | ns | 4.61±0.03 | 4.54±0.12 | ns |
| Body weight | 2350.00±67.69 | 2198.00±135.26 | ns | 1919.00±47.67 | 1862.22±37.37 | ns |
| Slaughter weight | 2051.00±53.76 | 2001.00±86.15 | ns | 1707.00±42.74 | 1635.56±30.14 | ns |
| Half-eviscerated weight | 1923.00±49.62 | 1859.00±80.12 | ns | 1545.80±45.70 | 1517.78±29.05 | ns |
| Eviscerated weight | 1521.00±40.56 | 1516.00±79.07 | ns | 1295.00±35.75 | 1264.44±25.93 | ns |
| Breast muscle weight | 261.71±13.78 | 259.31±11.50 | ns | 242.00±5.77 | 224.71±11.48 | ns |
| Leg muscle weight | 323.42±11.67 | 312.05±15.22 | ns | 257.53±7.33 | 247.49±9.89 | ns |
| Abdominal fat weight | 36.01±4.32 | 31.67±3.49 | ns | 44.28±3.98 | 43.54±5.91 | ns |

Comparisons between different comb sizes were performed using independent sample t-tests. Data are expressed as mean ± SEM, ns indicates no significance.
